# Characteristics of Carbon Material Formation on SBA-15 and Ni-SBA-15 Templates by Acetylene Decomposition and Their Bioactivity Effects

**DOI:** 10.3390/ma9050350

**Published:** 2016-05-09

**Authors:** Hsiu-Mei Chiang, Kuan-Yu Cho, Li-Xuan Zeng, Hung-Lung Chiang

**Affiliations:** 1 Department of Cosmeceutics, China Medical University, Taichung 40402, Taiwan; hmchiang@mail.cmu.edu.tw; 2 Department of Health Risk Management, China Medical University, Taichung 40402, Taiwan; maybe_cho@hotmail.com (K.-Y.C.); polaris962@gmail.com (L.-X.Z.)

**Keywords:** Santa Barbara Amorphous (SBA-15), mesoporous silica, polyaromatic hydrocarbons (PAHs), human lung cancer cell-A549, reactive oxidative stress (ROS)

## Abstract

Carbon spheres and tubes were formed from acetylene decomposition on SBA-15 and Ni-SBA-15 at 650–850 °C. At 650 °C, the decomposed carbons covered the surface of the support, and no carbon spheres and filament materials were formed. Carbon sphere formation occurred at 750 °C–850 °C; with diameters ranging from 0.8 μm–1.1 μm. For Ni-SBA-15, the diameters of the spheres and filaments were 0.8 μm and 62 nm, respectively, at 650 °C. At 750 °C, the diameter of the ball carbon materials ranged from 0.7 μm–0.8 μm, the diameter of the carbon tubes formed was 120–130 nm, and their pore diameter was 8.0 nm–11 nm. At 850 °C, the diameters of ball carbon materials and carbon tubes were similar to those of the materials at the formation temperature, 750 °C. Si, O and C were the main constituents of SBA-15; Ni-SBA-15 and carbon material formation supports. High-ring PAHs (such as BaP (five rings); IND (six rings); DBA (five rings) and B[*ghi*]P (six rings)) exist in carbon materials. SBA-15 revealed insignificant cytotoxicity, but Ni-SBA-15 inhibited the proliferation of human lung cancer cells (A549). Less inhibition on cell viability and reactive oxidative species (ROS) generation on A549 were determined for carbon material formation on the Ni-SBA-15 compared to the Ni-SBA-15.

## 1. Introduction

Nanotechnology engineering has given rise to the rapid development of many novel applications for sub-micron materials. The unique properties of materials at this size scale have spawned the fields of nanoscience and nanotechnology in various industries. Through manufacturing by-products and consumer use, these materials are then released into the environment and even into the human body [1,2]. The increasing use of nanomaterials has given rise to concerns about the potential risks and health hazards associated with exposure. Toxicological effects of nanomaterials are now intensively investigated in both *in vivo* and *in vitro* systems [3,4,5,6].

Carbon-based materials are commonly used among nanomaterials. Biological responses to carbon nanotubes (CNTs) are affected by multiple properties that include length, shape (single-wall or multiwall), fibrous surface area, aspect ratio and aggregability with or without the involvement of dispersion media [7,8,9,10,11,12,13,14,15].

Impurities, such as catalysts and polycyclic aromatic hydrocarbons (PAHs), are introduced into CNTs by the production process. These impurities have intrinsic toxicities, and their interaction with CNTs in cells can be cytotoxic [16,17,18,19].

Cytotoxicity, cytokine production and oxidative stress occur when various types of cells are cultured with CNTs [20,21,22,23,24,25]. CNTs cause cytotoxicity in mesothelial cells and FEi-Muta™ mouse lung epithelial cells through generation of reactive oxidative species (ROS)and downstream signal transduction such as mitogen-activated protein kinase [22,23]. One of the first target organs of nanoparticle exposure is the lung, which is directly exposed after inhalation of contaminated air. Pulmonary exposure to CNTs has caused rats and mice to develop fibrosis, granulation and inflammation in their lungs [26,27,28]. Due to their size, nanoparticles are distributed throughout the entire respiratory tract and can reach pulmonary alveoli [29]. Both single-walled carbon nanotubes (SWCNTs) and multiple-walled carbon nanotubes (MWCNTs) can pose potential health problems as described by extensive reports in the literature. Pulmonary effects of CNTs have been evaluated by a number of *in vivo* and *in vitro* studies.

Carbon monoxide, methane, acetylene and ethylene are common carbon sources to decompose over Fe, Co and Ni catalysts in the preparation of carbon nanomaterials [30,31,32]. The simple hydrocarbons can be effective for the synthesis of carbon materials, and alkenes can enhance the formation rate of CNTs. Therefore, acetylene was selected as the carbon source of carbon nanomaterials in this study. Nickel is highly active and selective in organic reactions because of its high capability to break C-C bonds [33], which is the main reason that nickel was chosen as the catalyst in this work. SBA-15 is hydrothermally stable, and its physicochemical properties were consistent throughout the highly ordered hexagonal mesopores [34,35,36]. Thermal chemical vapor deposition (CVD) was employed in this study. It has several advantages, including high purity, high yield, selective growth, and vertical alignment, and it is used to produce many carbon materials such as tubes and filaments [37], spheres [38], helices [39], bamboo, and onion [40,41].

This study investigated the decomposition of C_2_H_2_ on SBA-15 and Ni as a catalyst on SBA-15 (Ni-SBA-15) to form carbon materials by the CVD method. In addition, scanning electron microscopy (SEM), transmission electron microscopy (TEM), energy dispersive spectroscopy (EDS), Raman scattering analysis, and pore structure analysis were used to analyze the physicochemical properties of carbon materials. The human lung cancer cell A549 was used to determine the inhibition effects and reaction oxidative stress of SBA-15, Ni-SBA-15 and carbon material formation on SBA-15 and Ni-SBA-15.

## 2. Experimental

### 2.1. Material Preparation

#### 2.1.1. Santa Barbara Amorphous (SBA-15) Preparation

SBA-15 is synthesized under acidic conditions, using the amphiphilic tri-block copolymer as a structure-directing agent and by a hydrothermal method. The synthesis conditions (e.g., pH, block copolymer and hydrothermal temperature) can be varied widely, leading to various textural properties while keeping the framework unchanged.

Four grams of tri-block copolymer poly(ethylene glycol)-block-poly(propylene glycol)-block-poly(propylene glycol) (Pluronic P123, molecular weight 5800, EO_20_PO_70_EO_20_, Aldrich, St. Louis, MO, USA) was completely dissolved in a solution of 160 mL 2M HCl and mixed for 1 h. Then, 6.4 g of tetraethyl orthosilicate (TEOS, Aldrich, St. Louis, MO, USA) was added to the reaction mixture, and the mixture was stirred for 24 h at 30 °C. After the temperature was increased to 90 °C, the mixture was stirred, and the temperature was maintained for 24 h. The solid products were collected by filtration, thoroughly washed with distilled water, and dried overnight in air. The structure-directing agent was removed by calcination in air at 500 °C for 5 h. After cooling to room temperature, the particles were ground into SBA-15 powder.

#### 2.1.2. Nickel (Ni)-SBA-15

Nickel nitrate hexahydrate (Ni(NO_3_)_2_·6H_2_O, Aldrich, St. Louis, MO, USA) was used as the source of nickel. Ten grams of SBA-15 powder was impregnated with 10 mL distilled water solution containing 0.2476 g Ni(NO_3_)_2_·6H_2_O. The Ni(NO_3_)_2_ and SBA-15 mixture was placed into a sonicator to mix for 30 min at room temperature and filter out the solid materials, and then dried in an oven at 60 °C to remove the water. The dried Ni/SBA-15 particles were put into an electrical thermal furnace at a heating rate of 5 °C/min to 550 °C for 5 h. This process was employed to prepare 5% Ni-SBA-15.

#### 2.1.3. Carbon Material Preparation

The SBA-15 or Ni-SBA-15 was reduced under hydrogen atmosphere (200 mL/min) at a heating rate of 5 °C/min to reach 450 °C and maintained for 1 h in a CVD furnace. Acetylene was selected as the carbon source and decomposed on the SBA-15 and Ni-SBA-15 support to form carbon materials. Carbon materials were formed at 650, 750 and 850 °C for 30 min with a C_2_H_2_/N_2_ mixture at a flow rate of 200 mL/min (the volume ratio of N_2_:C_2_H_2_ was 3:1).The carbon material formation on SBA-15 template was presented as SBA-650, SBA-750 and SBA-850 for acetylene decomposition temperatures 650 °C, 750 °C and 850 °C, respectively. Acetylene decomposition on the Ni-SBA-15 template was presented as Ni-SBA-650, Ni-SBA-750, and Ni-SBA-850 for the various temperatures.

### 2.2. Characterization of Materials

#### 2.2.1. Scanning Electron Microscopy (SEM)

The surfaces of SBA-15, Ni-SBA-15 and decomposed carbon were examined using a JEOL JSM-6700F field emission SEM (Peabody, MA, USA)operated at 1–2 kV.

#### 2.2.2. Transmission Electron Microscope (TEM)

A high-resolution transmission electron microscope (HR-TEM, JEOL JEM-2010, Tokyo, Japan) was employed to examine the visualized structure of materials and operated at an accelerated voltage of 200 kV.

#### 2.2.3. Pore Size Distribution

Physical characteristics of the materials including specific surface area, micropore area, total pore volume, micropore volume, pore size distribution and pore diameter, were measured with N_2_ (g) adsorption (ASAP 2010 Pore Structure Analyzer, Micromeritrics Inc., Norcross, GA, USA) at 77 K with liquid N_2_. The BET surface area, micropore surface area, total pore volume, and micropore volume were calculated by the BET method [42], the BET surface area minus external surface area, the BJH method [43], the t-plot method and the Harkins-Jura method [44,45]. Standard materials including silica-alumina (surface area: 215 ± 6 m^2^/g, total pore volume: 0.61 ± 0.08 cm^3^/g, average pore diameter: 114 ± 15 Å), alumina (multipoint specific surface area 0.51 ± 0.03 m^2^/g) and molecular sieve (median pore diameter: 8.3 ± 0.2 Å) were obtained from Micromeritrics and employed for quality assurance and quality control.

#### 2.2.4. Dynamic Light Scattering (DLS) and Zeta Potential

The size of the particles in the supplemented cell medium was also measured by DLS using a Malvern Zetasizer Nano-ZS particle analyzer (Malvern Instruments, Southborough, MA, USA). The particle size was calculated from the Brownian motion of the particles using the Stokes-Einstein equation. Note that the particle size measurement on this instrument is based on the assumption that the particles are spherical to determine the motion in the solute as the actual particle.

A Malvern Zetasizer Nano ZS instrument (Malvern Instruments Ltd., Malvern, UK) was used to measure zeta potential at 25 °C for all samples. Samples prepared for the DLS measurements were loaded into a pre-rinsed folded capillary cell for the zeta potential measurements. A minimum of three measurements was made per sample.

### 2.3. Chemical Compositions

#### 2.3.1. Energy Dispersive Spectroscopy (EDS)

The surface composition of the material samples was analyzed with a TEM (JEOLJEM-2010, Tokyo, Japan) equipped with an energy dispersive X-ray spectrometer (EDS, DX-4 attached to the JEOL-2010, Tokyo, Japan).The analyzed elements included C, O, Si, and Ni. Analysis was performed on five samples in duplicate for quality assurance and control.

#### 2.3.2. Elemental Constituent

The tested materials were digested with a mixture of HNO_3_:HClO_4_:HF in a 3:5:2 proportion. A Perkin Elmer OPTIMA 3000 ICP-AES (PerkinElmer Inc., Waltham, MA, USA) was used to determine the Al, Ca, Fe, K, Mg, Na, Co, and Zn concentrations. Additionally, a SCIEX Elan Model 5000ICP-MS (PerkinElmer Inc., Waltham, MA, USA) manufactured by Perkin-Elmer was employed to determine the As, B, Ba, Be, Bi, Cd, Cr, Cu, Ga, In, Li, Mn, Mo, Ni, Pb, Sb, Se, Sr, and V concentrations.

#### 2.3.3. Raman Scattering Spectrometer

A Micro-Raman System (3D Nanometer Scale Raman PL Microspectrometer, Raman, Tokyo Instrument, Inc., Tokyo, Japan) using an Ar laser beam with a wavelength of 632.8 nm was used to analyze the microstructure of the nanotubes. The quality was identified by Raman spectroscopy using the 514 nm line of an argon laser operated at a laser power of 50 mW.

#### 2.3.4. Polycyclic Aromatic Hydrocarbon (PAH) Analysis

The 16 USEPA PAH standards (purity of >99%) were purchased from Supelco Inc. (Supelco Inc., Bellefonte, PA, USA).They are classified by the numbers of aromatic rings as follows: 2-ring including naphthalene (NaP); 3-ring including acenaphthylene (AcPy), acenaphthene (Acp), fluorene (Flu), phenanthrene (PA) and anthracene (Ant); 4-ring including fluoranthene (FL), pyrene (Pyr), benzo[*a*]anthracene (BaA) and chrysene (CHR); 5-ring including benzo[*b*]fluoranthene (BbF), benzo[*k*]fluoranthene (BkF) and benzo(a)pyrene (BaP); 6-ring including indeno[1,2,3-cd]pyrene (IND), bibenzo[*a*,*h*]anthracene (DBA), andbenzo[*ghi*]perylene (B[*ghi*]P). Dichloromethane, hexane, silica gel (0.063–0.200 mm) and other reagents were pesticide analysis grade and/or residue analysis grade and purchased from E. Merck (Merck KGaA, Darmstadt, Hesse, Germany).

The mixed solvent (Hexane:Dichloromethane = 50:50, by volume) was utilized to prepare the stock solutions. Blank analyses were performed regularly to ensure that no PAHs were present in laboratory reagents or the atmosphere.

Samples were extracted using the Soxhlet extraction procedure. The extract was concentrated on a rotary evaporator (EYELA, Tokyo, Japan) equipped with a water bath held at 40 °C, then reduced to 1 mL–2 mL. The extract was cleaned with solvent eluting in a silica column, through which 10 mL of hexane and 50 mL of the mixed solvent were respectively passed. The final solution (2 mL) was analyzed with the PAH gas chromatography (GC) method.

The GC apparatus (Agilent Technologies, Inc., Santa Clara, CA, USA) consisted of a Hewlett-Packard GC 6890 equipped with a mass (5973N) and split/splitless injector. An HP-5MS capillary column (Agilent Technologies, Inc., Santa Clara, CA, USA) (30 m at 0.32 mm i.d. with 0.25 μm film thickness) was used. Five concentrations of mixed standard solution were used to establish calibration curves for PAH measurement. The PAH spike was added to the blank sampling thimbles prior to extraction for recovery analyses. The average recoveries of PAHs based on QA/QC purpose ranged from 68% (naphthalene) to 97% (Benzo[*k*]fluoranthene).

### 2.4. Bioactivity

#### 2.4.1. Cell Viability Assay

The MTT assay was used to measure cell viability as previously described [46]. Human bronchoalveolar carcinoma A549 cell line was obtained from the American Type Culture Collection (Manassas, VA, USA). Briefly, A549 cells were cultured at a cell density of 5 × 10^4^ cells per well in 24-well plates per 1 mL medium. After 24 h, the cells were treated with 1 mL of various concentrations of carbon materials suspended in Dulbecco’s Modification of Eagle’s Medium (DMEM) for 24 h. In addition, some particulate aggregation was observed in the cell media. Therefore, sonication was conducted to reduce the aggregation of particles before they were added into the cells [47,48]. The cytotoxicity of carbon materials was evaluated in the cells cultured. Then, 500 μL of MTT (5 mg/mL in PBS) solution was added to each well. After 3 h of incubation, 1 mL of sodium dodecyl sulfate (SDS) solution in 0.01 N HCl was added to dissolve the formazan crystal produced in the cells. The absorbance of each well was then read at 570 nm using a microplate reader (Tecan, Grodig, Austria).

The inhibition rate of carbon materials was calculated using the following equation:

(1) $$\text{Inhibition}(\%)=\frac{\text{OD}570\text{ sample}}{\text{OD}570\text{ control}}\times 100$$

#### 2.4.2. Fluorescence Assay of Intracellular Reactive Oxidative Species (ROS)

The fluorescence assay was performed as previously described with minor modifications [49]. Briefly, the assay is based on the use of an established nonfluorescent (DCFDA)/fluorescent (DCF) system that measures ROS. ROS are in turn responsible for the generation of fluorescence. For the fluorescence assay, human lung cells (A549) were maintained in DMEM supplemented with 10% FBS, 100 U/mL penicillin, and 100 U/mL streptomycin at 37 °C in 5% CO_2_ humidified air. The cells were seeded at a density of 5 × 10^4^ cells/well in a 24-well plate for 24 h. After that, PBS was removed, and then various concentrations of carbon materials that had been prepared in serum-free DMEM were added and then incubated at 37 °C for 24 h. The cells were rinsed twice with 0.5 mL PBS and then incubated at 37 °C for 30 min in the presence of 10 μM DCFDA that had been prepared in DMEM. Next, the DMEM was removed, and the cells were washed twice with 0.5 mL PBS. The cells were then covered with 0.5 mL PBS. Images were observed under a fluorescence microscope (Leica DMIL, Wetzlar, Germany), and the fluorescence (emission 488 nm, excitation 520 nm) was measured using a microplate reader (Thermo Electron Corporation, Vantaa, Finland).

(2) $$\text{Relative fluorescence }\left(\%\right)=\left(\frac{A_{\mathrm{control}}-A_{\mathrm{sample}}}{A_{\mathrm{control}}}\right)\times 100$$

### 2.5. Statistical Analysis

Differences between groups were analyzed by ANOVA followed by the Scheffe’s test. *P* value < 0.05 was considered statistically significant.

## 3. Results and Discussion

### 3.1. Microscopic Photograph

#### 3.1.1. SEM Analysis

**Figure 1:**
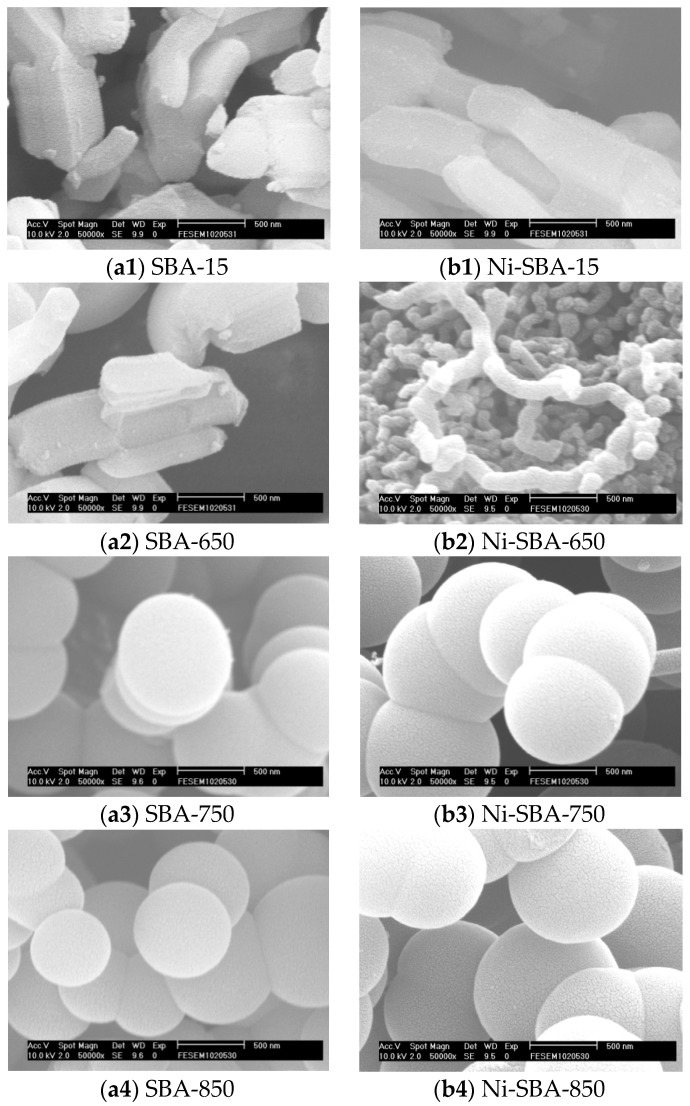
SEM photos of SBA-15, Ni-SBA-15 and carbon material formation on: (**a1**) SBA-15; (**a2**) SBA-650; (**a3**) SBA-750 and (**a4**) SBA-850; (**b1**) Ni-SBA-15; (**b2**) Ni-SBA-650; (**b3**) Ni-SBA-750 and (**b4**) Ni-SBA-850.

SEM micrographs of SBA-15 (Figure 1(a1)) reveal bundled rope-like units for SBA-15 and its derivatives with 5 wt % Ni (Figure 1(b1)). All SEM images had an ordered hexagonal structure for typical SBA-15 and Ni-SBA-15. The uniformly bundled macroscopic structure was mostly conserved after high-temperature thermal treatment, again reflecting the thermal stability of as-synthesized SBA-15 with metal loadings and thermal treatment. The rope-like hexagonal structure was about 0.3 μm in diameter and 1.0 μm in length for SBA-15. There were insignificant differences in the structures of Ni-SBA-15 compared with SBA-15. At 650 °C (Figure 1(a2)), some carbon-like soot covered the surface of SBA-15, and little spherical carbon formation occurred after the acetylene decomposition. The spherical carbon materials were formed at 750 °C (Figure 1(a3)) and 850 °C (Figure 1(a4)), and their diameters were 0.5 μm–0.9 μm and 0.35 μm–0.9 μm, respectively. However, there was an insignificant difference in the diameters of the spherical carbon materials.

For Ni-SBA-15 support, some filament-shaped carbon materials were formed at 650 °C (Figure 1(b2)). At 750 and 850 °C (Figure 1(b3,b4)), spherical and filament carbon materials were formed on the support, but the spherical carbon materials were in the majority. Some spherical chains were observed, and their diameters were 0.8 μm–1.0 μm for both 750 °C and 850 °C. In addition, the filament diameter was in the vicinity of 150 nm.

#### 3.1.2. TEM Analysis

**Figure 2:**
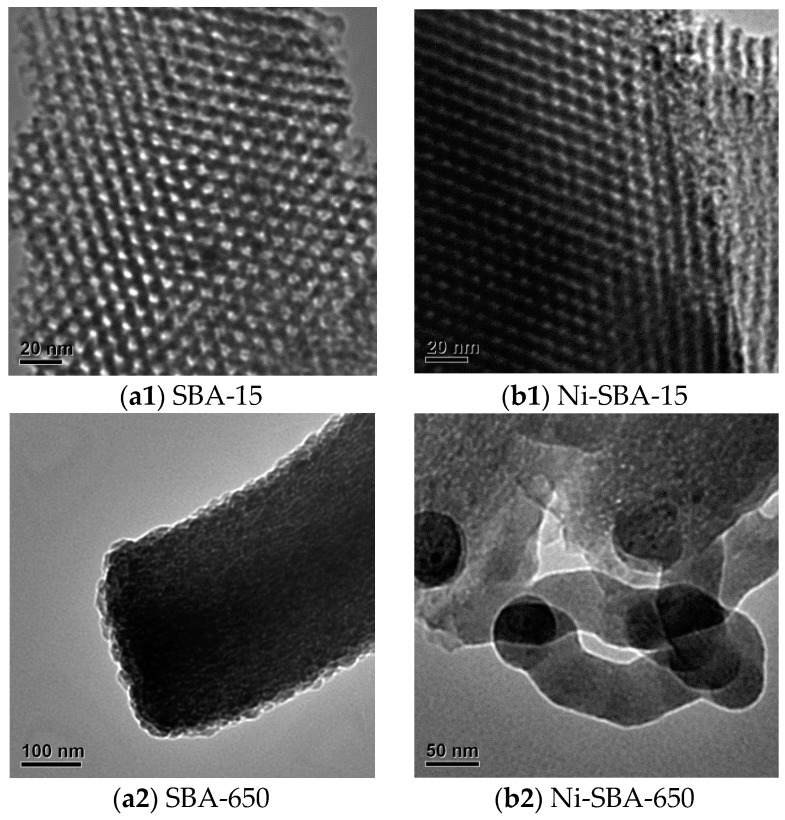

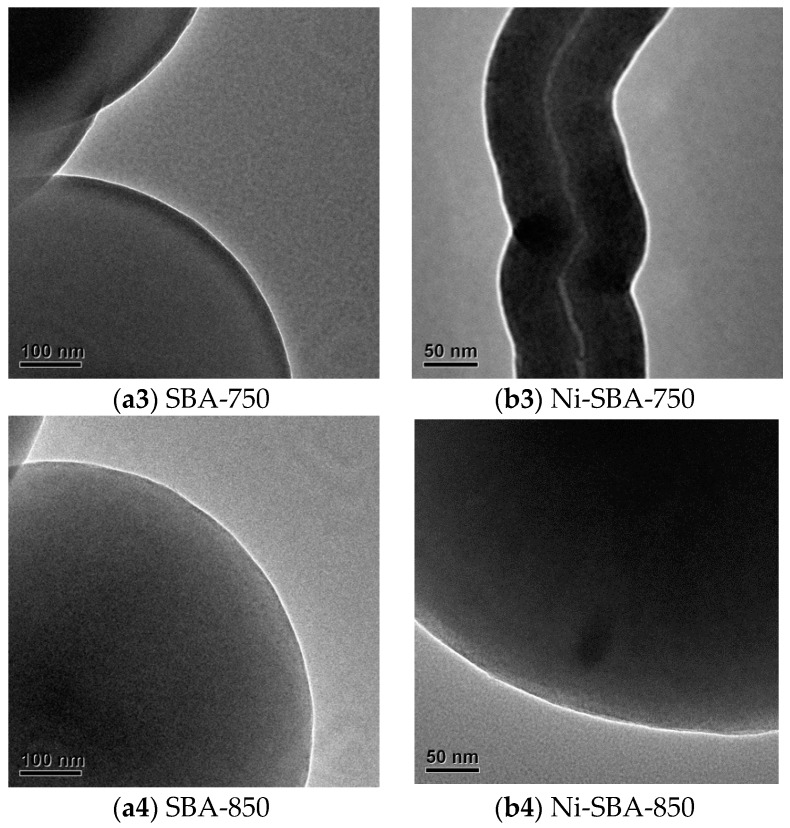
TEM photos of SBA-15, Ni-SBA-15 and carbon material formation on: (**a1**) SBA-15; (**a2**) SBA-650; (**a3**) SBA-750 and (**a4**) SBA-850; (**b1**) Ni-SBA-15; (**b2**) Ni-SBA-650; (**b3**) Ni-SBA-750 and (**b4**) Ni-SBA-850.

Figure 2 shows that the diameters of rope-like units were in the range of 0.2 μm–0.5 μm for SBA-15 (Figure 2(a1)), with a slightly enlarged shape for Ni-SBA-15 (0.25 μm–0.71 μm) (Figure 2(b1)). The uniformly bundled macroscopic structure was mostly conserved after high-temperature thermal treatment, again reflecting the thermal stability of as-synthesized SBA-15 with metal loadings and thermal treatment.

The metal sites were evenly distributed for loadings of 5 wt %, and the considerable decrease in the surface area of the impregnated sample, Ni-SBA-15, can be attributed to either the loss of crystallites or pore blockage (Figure 2). The image of SBA-15 (Figure 2(2a)) and Ni-SBA-15 (Figure 2(2b)) shows a hexagonal mesoporous structure and regular-sized nickel particles distributed on the TEM images. There was no significant formation of filament or sphere shapes of carbon materials on the SBA-15 at 650 °C (Figure 2(a2)). Ball shape carbon materials were observed, and their diameters were 0.8 μm–1.1 μm and 0.8 μm–0.9 μm at 750 (Figure 2(a3)) and 850 °C (Figure 2(a4)), respectively. In addition, some nickel particles were determined in Ni-impregnated SBA-15. The size of nickel particles ranged from 10 μm–40 nm (Figure 2(b1,b2) in supplementary materials-Figures S2-a and S2-b). The diameters of the spheres and filaments were 0.8 μm and 62 nm, respectively, on Ni-SBA-15 at 650 °C (Figure 2(b2)). At 750 °C (Figure 2(b3)), the ball carbon materials had diameters of 0.7 μm–0.8 μm, and the carbon tubes formed had diameters ranging from 120 nm–130 nm, with pore diameters ranging from 8.0 nm–11 nm. Some nickel particles were observed at the end of the filaments. At 850 °C (Figure 2(b4)), the diameters of the ball carbon materials were in the vicinity of 0.8 μm–0.9 μm, and the diameters of the carbon tubes were similar to those of the materials formed at 750 °C, with the pore diameter down to 6 nm–9 nm.

### 3.2. Surface Area/Pore Size Distribution

**Figure 3:**
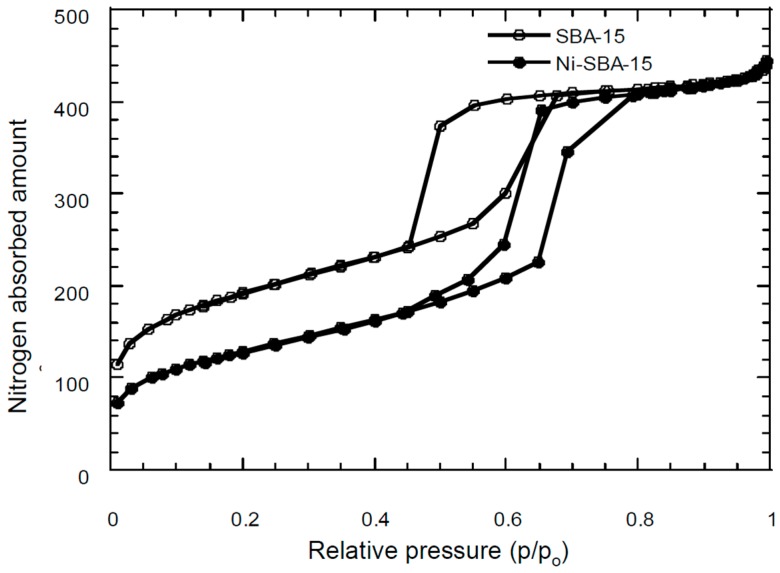
Nitrogen adsorption and desorption isotherms of SAB-15, and Ni-SBA-15.

**Figure 4:**
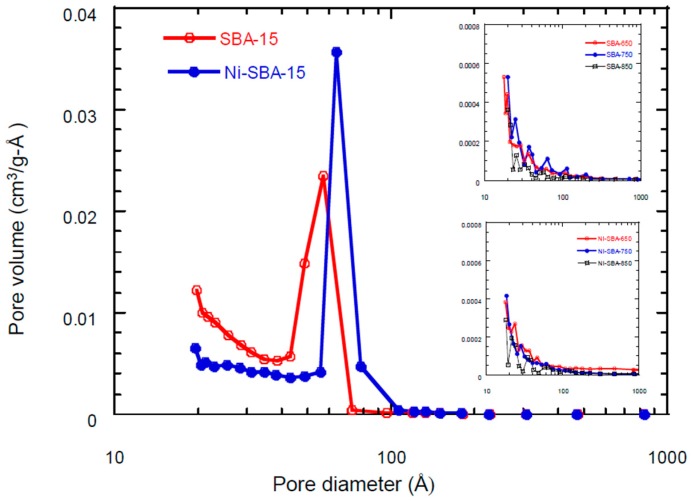
Pore size distribution of SAB-15, Ni-SBA-15 and carbon materials.

Nitrogen adsorption–desorption isotherms reveal typical type IV isotherms with H1-type hysteresis for SBA-15 and Ni-SBA-15 as shown in Figure 3. A hysteresis loop was observed indicating that the ink-bottle-shaped pores were present for both support materials. In addition, a narrow space of hysteresis was determined for Ni-SBA-15, which could be attributed to Ni impregnation and blockage of some micropores on the support to reduce hysteresis during nitrogen desorption. Table 1 shows the physical characteristics of carbon materials on SBA-15 and Ni-SBA-15. There was a 30% decrease of the specific surface area of Ni-SBA-15 compared with SBA-15. In addition, the pore diameter was 35% enlarged from 41.4 Å (SBA-15) to 55.9 Å (Ni-SBA-15). The peak of pore size distribution was shifted to the larger pores for Ni-SBA-15 (Figure 4).

The reduction of the specific surface area and pore volume of both supports after acetylene decomposition indicated that the carbon materials covered the surface of the support materials. At the decomposition temperature of 650 °C, the carbon materials on the Ni-SBA-15 revealed a higher surface area than the SBA-15. High decomposition temperatures revealed high specific surface area and total pore volume reduction, which could be due to the high carbon material yield on the surface of SBA-15 and Ni-SBA-15 at higher temperatures.

### 3.3. Raman Scattering Analysis

**Figure 5:**
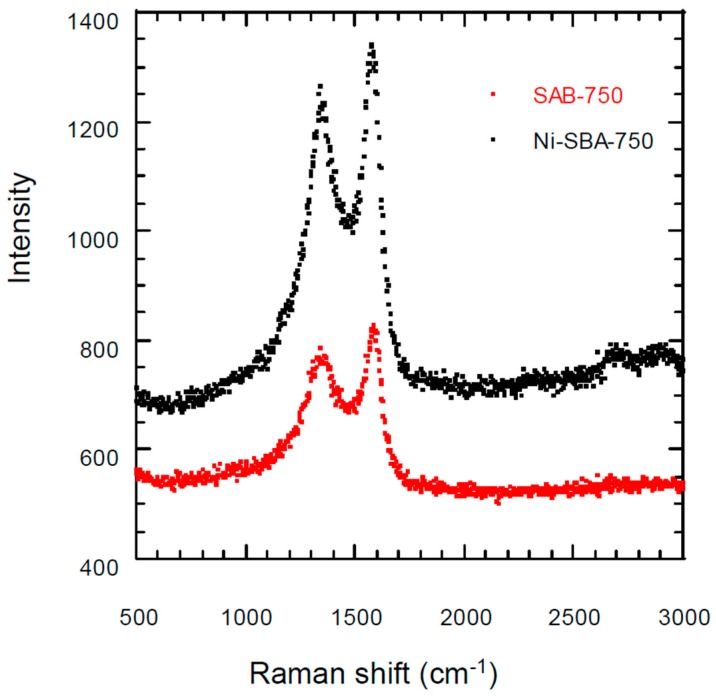
Raman spectra of typical carbon materials on SBA-15 and Ni-SBA-15 at 750 °C.

The graphite mode and the non-crystal structure consisting of the defects, lattice distortion and dislocation of the carbon structure were analyzed by the Raman spectrum. Two main peaks were determined for carbon materials, D-band (disorder mode, amorphous carbon) and G-band (graphite sp^2^ structure) were evidenced at 1350 and 1590 cm^−1^, respectively. The relative intensity ratio of D- and G-bands could explain the degree of graphitization and purity of carbon tubes. Raman spectrum curves for SBA-750 and Ni-SBA-750 (presented in Figure 5) showed two peaks: at 1350 cm^−1^ (D band) and 1600 cm^−1^ (G band). C_2_H_2_ was decomposed by the Ni catalyst to form much more sp^2^ graphite structure carbon materials than non-catalysts on SBA-15 support.

### 3.4. Element Constituents

#### 3.4.1. Main Elements

Table 2 shows the elemental constituents as determined by the EDS spectra for SBA-15, Ni-SBA-15, and carbon materials on supports at different temperatures. Silica and oxygen were the major elements in SBA-15, but there was some carbon content in the template (carbon content comes from the synthesis of raw materials). A total of 7.8% nickel was doped on Ni-SBA-15, which is different from the results of ICP-MS (5.7% in Table 3) due to the different analysis methods. For SBA-15, the carbon content ranged from 47%–62% and increased with decomposition temperature. The carbon content was about 58%–60% on the carbon material formation template Ni-SBA-15.

#### 3.4.2. Trace Elements

Sodium, zinc, potassium, aluminum, calcium, magnesium, and iron were abundant in SBA-15 and carbon material formation SBA-15 (shown as Table 3). Nickel concentrations were in the range of 6.5–21 μg/g for SBA-15 and its carbon materials. After nickel impregnation, the nickel concentration could be as high as 57,100 μg/g in Ni-SBA-15. The concentration of some metals, e.g., zinc, cobalt and indium, were increased in Ni-SBA-15 compared to SBA-15, which could be attributed to impurities associated with the Ni-impregnation process.

### 3.5. PAH Content of Carbon Materials

**Figure 6:**
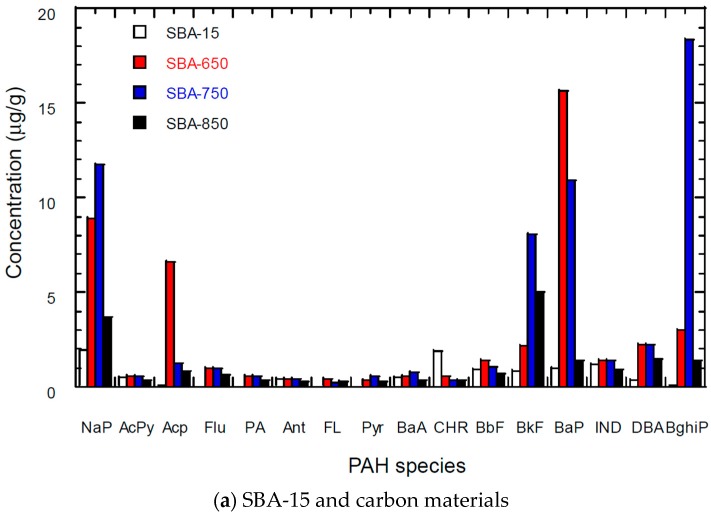

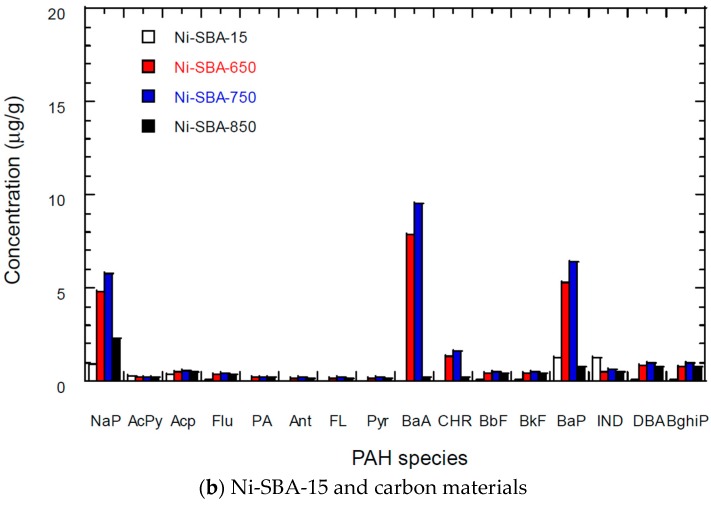
PAH content in the SBA-15, Ni-SBA-15, carbon materials on SAB-15 and Ni-SBA-15 at different decomposition temperatures. (**a**) SBA-15 and carbon materials and (**b**) Ni-SBA-15 and carbon materials.

Figure 6 shows the PAH content in the SBA-15, Ni-SBA-15, and acetylene decomposition on SBA-15 and Ni-SBA-15. The content of 16 PAHs was 9.6 μg/g for SBA-15. The contents of 16 PAHs of acetylene decomposition to form carbon materials were 46 μg/g at 650 °C, 60 μg/g at 750 °C and 18 μg/g at 850 °C. For Ni-SBA-15, the PAH content was 4.5 μg/g. The PAH content was 24, 29, and 8.1 μg/g for Ni-SBA-650, Ni-SBA-750, and Ni-SBA-850, respectively. Benzo[*a*]pyrene (five rings), naphthalene (two rings), acenapthylene (three rings), benzo[*ghi*]perylene (six rings), benzo[*k*]fluoranthene (five rings), dibenz[*a*,*h*]anthracene (five rings), indeo[1,2,3-cd]pyrne (six rings), and benzo[*b*]fluoranthene (five rings) were the major PAHs, contributing 65%–92% of PAHs in SBA-15 and SBA-15 carbon materials.

Benz[*a*]anthracene (four rings), benzo[a]pyrene (five rings), naphthalene (two rings), benzo[*ghi*]perylene (six rings), dibenz[*a,h*]anthracene (five rings), indeo[1,2,3-cd]pyrene (six rings), benzo[*b*]fluoranthene (five rings), and chrysene (four rings) were the major PAHs, contributing 74%–91% of PAHs in Ni-SBA-15 and Ni-SBA-15 carbon materials.

Based on the spherical and filament carbon materials formed on the SBA-15 and Ni-SBA-15, most materials are the sp^2^ structure; therefore, there were few impurities such as PAHs in the carbon materials. At this point, semi-fullerenes form on the curved catalyst surface and then grow into carbon nanotubes [50]. Kroto *et al.* [51] indicated that PAHs with five- and six-membered rings are abundant. Three- and four-membered rings are very unstable, which could be a reason for the abundance of high-ring PAHs in the carbon materials.

The Ni-impregnated template (Ni-SBA-15) catalyzed to decompose acetylene and thereby form more carbon materials than the non-Ni template (SBA-15). Acetylene can decompose to form PAHs and carbon materials following the hydrogen abstraction/C_2_H_2_ addition mechanism [52]. The nickel catalyst could be a reason for the low PAH content in the carbon materials of Ni-SBA-15. A high fraction of PAHs was found in the high-ring PAHs. BaP (five rings), IND (six rings), DBA (five rings) and B[*ghi*]P (six rings) could be the precursors of carbon materials.

PAH formation and growth occur by reactions with single aromatics, other PAHs, and acetylene [53]. Subsequent nucleation or inception forms small soot particles, and the coagulation and mass addition from gas phase species and carbonization of the particulate matter can lead the soot growth to form carbon materials such as spheres and filaments.

### 3.6. Particle Size and Surface Charge

The effective particle size was measured with DLS and is thus equivalent to the hydrodynamic radius of the particles. The average particle size ranged from 2 μm–6 μm for SBA-15, Ni-SBA-15, and carbon material formation SBA-15 and Ni-SBA-15 (shown as Table 4). The size did not depend on the pyrolysis temperature. It is important to note that the same nanomaterial might display different results in cell studies; even if the nanoparticles display a certain size after synthesis, during the *in vitro* and *in vivo* studies they might aggregate into vastly different shapes and sizes, which may dictate the outcome and interpretation of results [54].

The physicochemical properties (such as size, surface properties and crystal phase) of nanoparticles can affect their toxic potential [55,56,57,58]. Particle size and surface properties played key roles in the cellular uptake as an adhesion process followed by an internalization process for nanoparticles [59,60]. The interaction of particles with cells is known to be strongly influenced by particle size, shape and surface chemistry on cellular internalization and intracellular trafficking [61]. They indicated the internalization of non-spherical particles with dimensions as large as 3 μm. For negative charge surface particles, the weakened electrostatic repulsion forces were between particles and the cell membranes, and the surface charge was dependent on the cellular uptake [62]. Positively charged surface particles exhibited a stronger affinity for the negatively charged cell membrane, accounting for its higher cellular uptake [59,62]. However, cellular uptake of particles can be affected not only by surface charge but also by other physicochemical characteristics (such as size, shape, constituents, functional groups, toxicity *etc.*).

The SBA-15, Ni-SBA-15, and carbon materials formed on SBA-15 and Ni-SBA-15 were analyzed for surface charge using zeta potential measurement at pH 7. A negative surface charge was determined in SBA-15, Ni-SBA-15, and carbon material formation in SBA-15 and Ni-SBA-15. As the zeta potential decreased, the repulsive forces between particles increased. However, in terms of zeta potential, the results indicated that there was no significant difference between SBA-15 and carbon material formation SBA-15. Generally, particle surfaces play an important role in toxicity as they make contact with cells and biological materials. There has been evidence of uptake of negatively charged particles despite the unfavorable interaction between the particles and the negatively charged cell membrane.

### 3.7. Bioactivity of Materials

#### 3.7.1. MTT (3-(4,5-Dimethylthiazol-2-yl)-2,5-Diphenyltetrazolium Bromide) Assay

**Figure 7:**
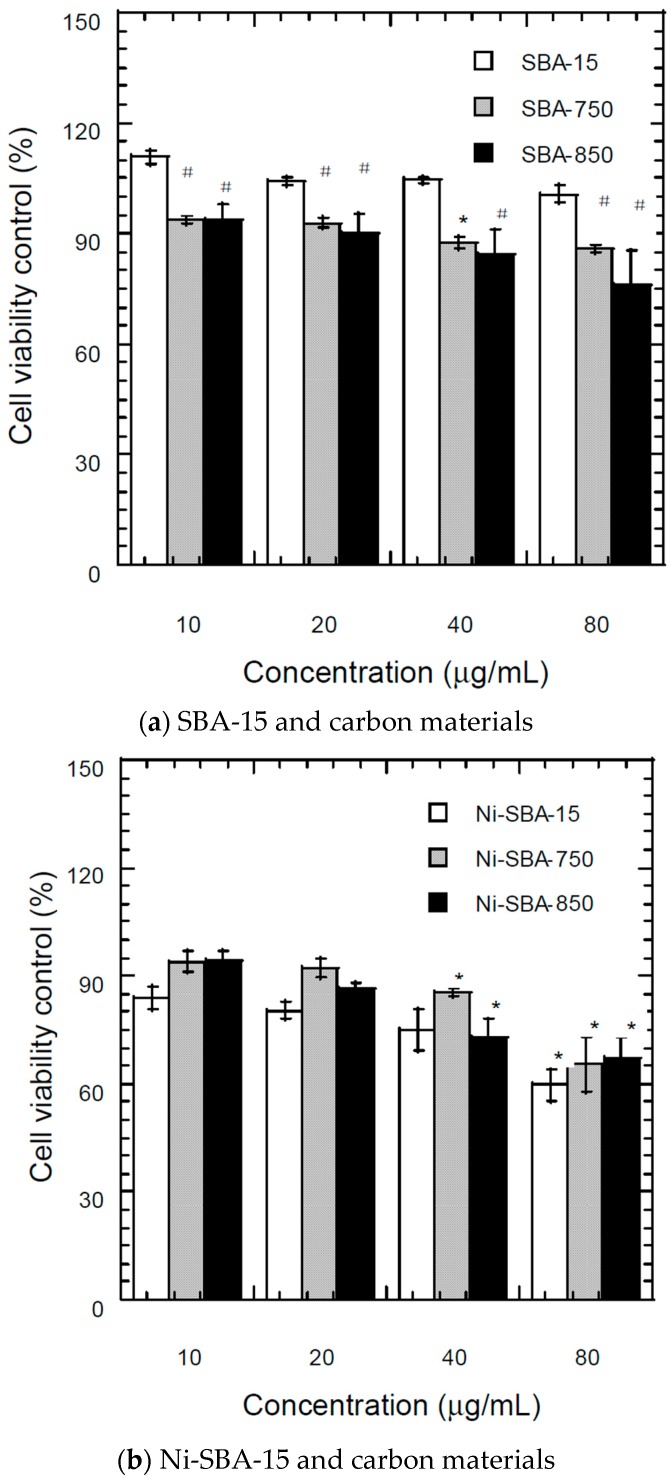
Cell viability in the cell culture medium after 24 h exposure to (10–80 μg/mL) of (**a**) SBA-15, and SBA-750, or SBA-850 and carbon materials; (**b**) Ni-SBA-15, and Ni-SBA-750, or Ni-SBA-850 and carbon materials. Values are mean ± SD from three independent experiments. Significance *versus* control group: * *p* < 0.05; # *p* < 0.05 compared to SBA-15.

MTT assay was used to determine the cytotoxicity of SBA-15, Ni-SBA-15 and carbon materials on A549 cells. According to the results of the MTT assay, SBA-15 exhibited an insignificant effect on cell viability at doses ranging from10 to 80 μg/mL (shown in Figure 7a). The cell viability of SBA-15 ranging from 10 to 80 μg/mL was 101% to 111% of the control (Figure 7a), which indicated that the SBA-15 did not inhibit the growth of the A549 cell. However, the cell growth was significantly inhibited by SBA-750 and SBA-850 at the same dose (Figure 7a). The cell viability was 86 to 94% for SBA-750 at doses from 10 to 80 μg/mL, and 76% to 92% for SBA-850.The cell viability was reduced as the dose of carbon materials increased. SBA-750 and SBA-850 exhibited significant inhibition of cell viability at doses greater than 10 μg/mL. As the results of the MTT assay indicated, carbon materials can inhibit cell viability.

For Ni-SBA-15 (Figure 7b), doses ranging from 10 to 80 μg/mL corresponded to cell growth from 60% to 84%. The cell viabilities decrease as the dose increases, and significant inhibition occurs at 80 μg/mL. Lower cell growth inhibition was determined on Ni-SBA-750 and Ni-SBA-850 compared with Ni-SBA-15.Ni-SBA-15, Ni-SBA-750 and Ni-SBA-850 exhibited significant inhibition of cell proliferation at 40 μg/mL (Figure 7b). In addition, compared with SBA-15, the cell viabilities of Ni-SBA-15 were lower than those of SBA-15. As Table 3 shows, the content of nickel decreased as the formation temperature of carbon materials increased. Nickel may be more toxic than carbon-based materials. The formation of carbon material on the surface of Ni-SBA-15 can reduce the exposure frequency of the cell on the Ni metal. TEM analysis also showed that when nickel is covered by carbon filaments, its toxicity is reduced.

Examining the influence of nanomaterial shape and size on cell interactions is crucial, as this can have implications for toxicity [63]. Wang *et al.* [64] indicated that C_60_ did not lead to a significant reduction of cell viability, but it presented an increase of intracellular reactive oxygen species. The neutral and negatively charged nanoparticles adsorbed much less on the negatively charged cell membrane surface and consequently showed lower levels of internalization compared to the positively charged particles [54].

#### 3.7.2. Fluorescence Assay of Intracellular ROS

**Figure 8:**
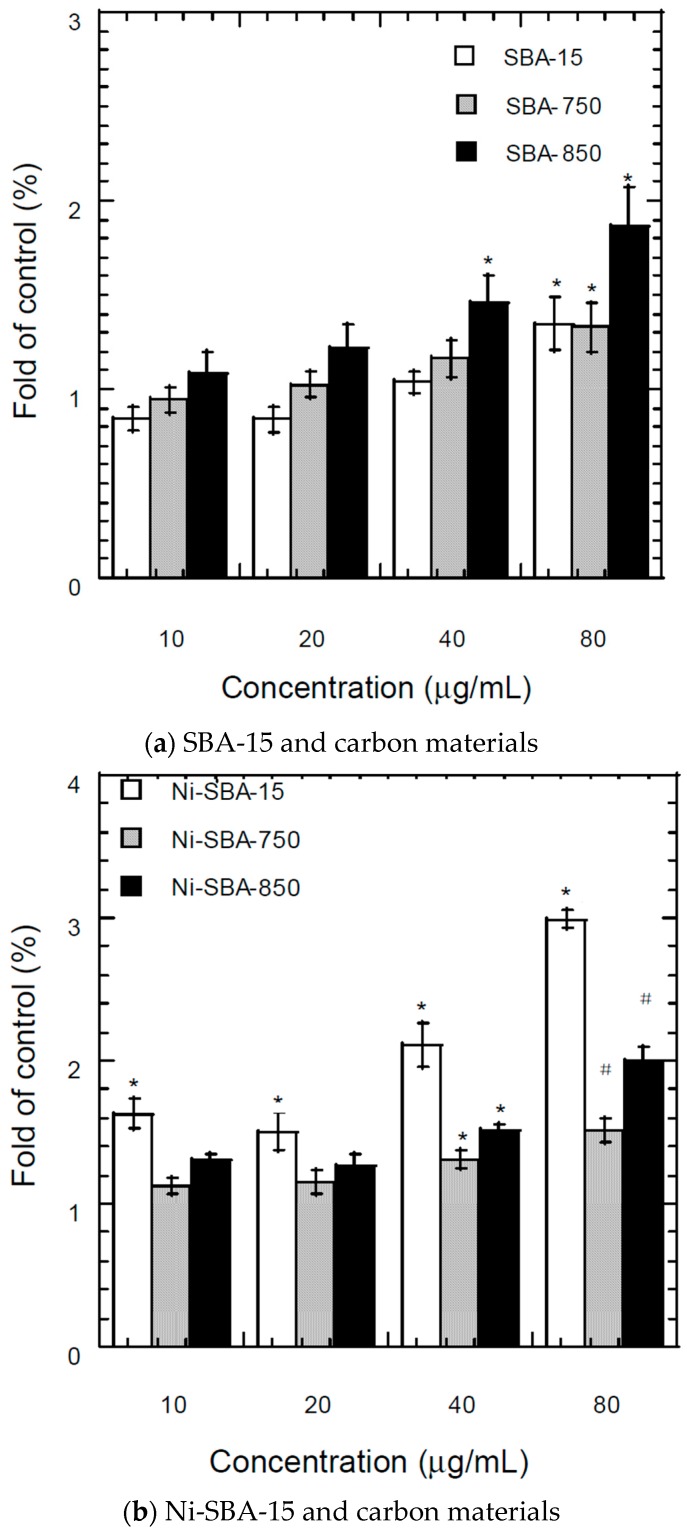
ROS in the cell culture medium after 24 h exposure to (10–80 μg/mL) of (**a**) SBA-15, and SBA-750, or SBA-850 and carbon materials; (**b**) Ni-SBA-15, and Ni-SBA-750, or Ni-SBA-850 and carbon materials. Values are mean ± SD from three independent experiments. Significance *versus* control group: * *p* <0.05; # *p* <0.05 compared to SBA-15.

DCFDA staining and fluorescence microscopy were used to qualitatively characterize the degree of ROS generation. A549 cells were exposed to 10 μg/mL to 80 μg/mL of SBA-15, Ni-SBA-15, and carbon material formation of SBA-15 and Ni-SBA-15 (Figure 8).

For carbon material formation on SBA-15, the ROS was increased with the increase of the exposure dose (Figure 8a). Carbon material formation at high temperature corresponded to high ROS and lower cell viability. ROS levels were markedly higher in particle exposure in the A549 cell than in control cells at particle levels higher than 80 μg/mL for SBA-15 and SBA-750, and higher than 40 μg/mL for SBA-850 (Figure 8a).

In addition, intracellular ROS generation of Ni-SBA-15, Ni-SBA-750 and Ni-SBA-850 was also determined. The ROS was 1.6, 1.5, 2.1 and 3.0-fold of that of the control at 10, 20 μg/mL, 40 μg/mL, and 80 μg/mL of Ni-SBA-15, respectively (Figure 8b). Ni-SBA-750 and Ni-SBA-850 induced significant ROS in A549 cells at 40 μg/mL (Figure 8b). The intracellular ROS of Ni-SBA-15 was higher than the same dose of SBA-15. The results indicated that the intracellular ROS generation of Ni-SBA-15 may contribute to the cytotoxicity of the material.

Based on the bioactivity, large surface area materials (such as SBA-15) did not inhibit the activity and generate the ROS to cause the cell death of A549. Generally, larger surface area led to increased reactivity and increased the source of reactive oxygen species in the *in vitro* experiments [55,65,66,67].

The Ni-impregnated SBA-15 could cause cytotoxicity and increase ROS (Figure 8b). Carbon materials were more likely to induce cell death and increase the ROS compared with SBA-15. For the carbon material formation of Ni-SBA-15, the carbon materials covered the support and nickel; therefore, low bioactivity effects were determined compared to the Ni-SBA-15. The toxicity of carbon materials could be caused by the PAH content, but the results of MTT and ROS did not address PAH content.

In this work, the cytotoxicity tests after the exposure of particles from carbon materials, silica templates and catalysts were only conducted for the lung cancer cell-A549. However, the cytotoxicity of particle exposure on normal cells such as lung epithelial cell could be an important issue to determine the biological effects. Particle exposure on normal cells will therefore become the focus of future work.

## 4. Conclusions

Carbon materials were formed on SBA-15 and Ni-SBA-15 during acetylene decomposition. The specific surface area was reduced after Ni impregnation on SBA-15. Carbon material formation on the surface of SBA-15 and Ni-SBA-15 significantly reduced the specific surface area and pore volume, and pore size distribution shifted to large pores, thereby enlarging the average pore diameter. benzo[*a*]pyrene (five rings), benzo[*ghi*]perylene (six rings), dibenz[*a,h*]anthracene (five rings), indeo[1,2,3-cd]pyrne (six rings), and benzo[*b*]fluoranthene (five rings) were the major polycyclic aromatic hydrocarbons (PAHs) in carbon material formation on supports during acetylene decomposition. High-ring PAHs could be the precursors of carbon materials. In this study, large surface area material (such as SBA-15) did not inhibit the activity and generate the ROS to cause the cytotoxicity of A549. Nickel-impregnated SBA-15 could inhibit cell activity compared to SBA-15. Carbon materials could inhibit cell growth and reflect on the MTT detection. However, carbon material formation on Ni-SBA-15 could reduce cell proliferation compared to Ni-SBA-15.This study applies information about the physicochemical characteristics and safety of carbon materials formed by various temperatures. It is useful for the production of optimum carbon materials with lower cytotoxicity.

## Figures and Tables

**Table 1:** Physical characteristics of carbon materials on SBA-15 and Ni-SBA-15.

Materials	SSA * (m^2^/g)	TPV (cm^3^/g)	PD (Å)	MSA (m^2^/g)	MPV (cm^3^/g)
SBA-15	652 ± 11	0.673 ± 0.013	41.35 ± 0.29	117.92 ± 4.81	0.062 ± 0.002
SBA-650	7.85 ± 1.73	0.02455 ± 0.001	124.975 ± 18.29	-**	-
SBA-750	9.57 ± 3.32	0.0222 ± 0.007	93.435 ± 4.43	-	-
SBA-850	4.884 ± 0.18	0.0126 ± 0.003	102.803 ± 18.80	-	-
Ni-SBA-15	472 ± 25	0.686 ± 0.003	58.2 ± 3.31	25.74 ± 4.74	0.0122 ± 0.002
Ni-SBA-650	11.737 ± 0.26	0.0762 ± 0.006	259.45 ± 14.59	-	-
Ni-SBA-750	5.8275 ± 2.62	0.0168 ± 0.005	91.36 ± 16.65	-	-
Ni-SBA-850	5.405 ± 0.93	0.0125 ± 0.004	120.03 ± 21.84	-	-

* SSA: BET specific surface area; TPV: total pore volume; PD: pore diameter; MSA: micropore surface area; MPV: micropore pore volume; **-: not detectable.

**Table 2:** Element constituents (%) of SBA-15, Ni-sba-15, and carbon materials with support at different temperatures by EDS analysis.

Material	C	O	Si	Ni
SBA-15	13.0 ± 4.6	36.7 ± 4.6	50.3 ± 8.4	-*
SBA-650	47.6 ± 0.5	24.4 ± 2.2	28.1 ± 1.7	-
SBA-750	60.3 ± 4.2	19.4 ± 2.6	20.3 ± 1.5	-
SBA-850	61.8 ± 3.1	15.2 ± 2.9	23.0 ± 0.2	-
Ni-SBA-15	19.8 ± 14.3	33.9 ± 2.9	37.5 ± 10.6	7.8 ± 2.5
Ni-SBA-650	57.8 ± 3.9	14.4 ± 1.2	22.9 ± 3.4	4.9 ± 0.7
Ni-SBA-750	60.4 ± 9.7	15.4 ± 3.0	19.9 ± 6.1	4.1 ± 0.6
Ni-SBA-850	60.0 ± 4.8	14.9 ± 0.6	21.4 ± 4.2	3.7 ± 1.2

*-: not detectable.

**Table 3:** Trace element constituents of SBA-15, Ni-SBA-15, and carbon material formation supports.

Element	SBA-15	SBA-650	SBA-750	SBA-850	Ni-SBA-15	Ni-SBA-650	Ni-SBA-750	Ni-SBA-850
Ni	21 ± 4.7	10 ± 3.2	8.3 ± 2.5	6.5 ± 3.1	57,100 ± 6543	45,860 ± 5325	36,500 ± 1549.1	23,470 ± 2974.2
Zn	153 ± 45.8	133 ± 50.3	105 ± 32.7	89 ± 45.6	201 ± 65.3	178 ± 84.1	125 ± 54.3	103 ± 47.7
Na	230 ± 119.4	175 ± 103.6	154 ± 85.1	72 ± 43.2	180 ± 89.5	154 ± 31.5	106 ± 37.9	86 ± 25.3
K	102 ± 21	89 ± 26.9	75 ± 17.6	64 ± 20.3	131 ± 34.5	116 ± 40.3	98.3 ± 17.9	82.5 ± 34.5
Al	95.2 ± 6.5	90.2 ± 10.1	87 ± 6.3	65 ± 5.4	86.3 ± 8.2	78.5 ± 9.1	68.9 ± 10.5	63.4 ± 7.8
Ca	43.1 ± 5.2	40.6 ± 8	35.3 ± 7.5	28.3 ± 3.4	57.3 ± 10.3	45.3 ± 4.5	38.7 ± 2.7	26.1 ± 5.2
Mg	50.2 ± 14.1	45.3 ± 18.9	40.6 ± 20.1	32.9±14.9	39.4 ± 12.5	38.6 ± 21.6	32.7 ± 11.5	24.6 ± 17.5
Co	12.3 ± 5.1	11.2 ± 5.2	9.7 ± 3.2	8.4 ± 4.2	37.8 ± 6.8	32.6 ± 6.7	28.5 ± 4.8	20.3 ± 3.1
Fe	45.1 ± 15.3	40.2 ± 6.7	38.7 ± 8.4	30.6 ± 4.9	37.4 ± 10.5	35.8 ± 5.3	30.2 ± 4.2	19.8 ± 1.6
In	<5.0	<5.0	<5.0	<5.0	7.29 ± 3.2	6.23 ± 3.6	5.87 ± 2.4	5.34 ± 2.1
B	<5.0	<5.0	<5.0	<5.0	5.57 ± 1.5	<5.0	<5.0	<5.0
Li	<5.0	<5.0	<5.0	<5.0	5.11 ± 0.76	<5.0	<5.0	<5.0
Cd	5.2 ± 1.5	<5.0	<5.0	<5.0	<5.00	<5.0	<5.0	<5.0

Mo, As, Cr, Pb, Ba, Cu, Sb, Be, Ga, Se, Bi, Mn, Sr, and V were less than method detection limit (<5.00 μg/g).

**Table 4:** Zeta potential and particle size of SBA-15, Ni-SBA-15 and carbon materials.

Materials	Zeta Potential (mV)	Particle Size (nm)
SBA-15	−16.2 ± 1.35	3568.7 ± 505.5
SBA-650	−18.9 ± 1.35	5966.7 ± 844.9
SBA-750	−14.9 ± 2.01	2023.7 ± 631.0
SBA-850	−15.1 ± 1.99	2399.3 ± 407.4
Ni-SBA-15	−20.8 ± 0.1	2896.3 ± 381.5
Ni-SBA-650	−6.2 ± 0.68	2280.4 ± 509.4
Ni-SBA-750	−21.9 ± 3.81	3430.0 ± 604.7
Ni-SBA-850	−11.8 ± 1.95	3843.7 ± 853.3

## Supplementary Materials

The following are available online at www.mdpi.com/1996-1944/9/5/350/s1, Figure S1-a: SEM photos of SBA-15, and carbon material formation on SBA-15; Figure S1-b: SEM photos of Ni-SBA-15 and carbon material formation on Ni-SBA-15; Figure S2-a: TEM photos of SBA-15, and carbon material formation on SBA-15; Figure S2-b: TEM photos of Ni-SBA-15 and carbon material formation on Ni-SBA-15.
