# Supplementary material for: Characteristics of Carbon Material Formation on SBA-15 and Ni-SBA-15 Templates by Acetylene Decomposition and Their Bioactivity Effects
Source: Materials (Basel). 2016 May 9;9(5):350. doi: 10.3390/ma9050350 (PMC5503084; doi:10.3390/ma9050350)
Supplement: Supplementary file 1 [file materials-09-00350-s001.pdf]

Supplementary Materials: Characteristics of Carbon Material Formation on SBA-15 and Ni-SBA-15 Templates by Acetylene Decomposition and Their Bioactivity Effects

Hsiu-Mei Chiang, Kuan-Yu Cho, Li-Xuan Zeng and Hung-Lung Chiang

1. SEM Scattering

The surfaces of SBA-15, Ni-SBA-15 and decomposed carbon were examined using a JEOL JSM-6700F field emission SEM (Peabody, MA, USA) operated at 1–2 kV.

Some SEM scattering photos were presented in Figures S1-a and S1-b. At 650 °C, some carbon-like soot covered the surface of SBA-15, and little spherical carbon formation occurred after the acetylene decomposition. The spherical carbon materials were formed at 750 °C (Figure S1-a(3-1,3-2)) and 850 °C (Figure S1-a(4-1,4-2)), and their diameters were 0.5–0.9 μm and 0.35–0.9 μm, respectively. There was an insignificant difference in the diameters of the spherical carbon materials.

For Ni-SBA-15 support, some filament-shaped carbon materials were formed at 650 °C (Figure S1-b(2-1,2-2)). At 750 °C (Figure S1-b(3-1,3-2)) and 850 °C (Figure S1-b(4-1,4-2)), spherical (0.8–1.0 μm in diameter) and filament (150 nm in diameter) carbon materials were formed on the support, but the spherical carbon materials were in the majority.

2. TEM Scattering

A high-resolution transmission electron microscope (HR-TEM, JEOL JEM-2010, Tokyo, Japan) was employed to examine the visualized structure of materials and operated at an accelerated voltage of 200 kV. The TEM scattering photos are shown in Figures S2-a and S2-b.

No filament or spherical shapes of carbon materials formed on the SBA-15 at 650 °C. Spherical carbon materials were observed, and their diameters were 0.8–1.1 μm and 0.8–0.9 μm at 750 °C (Figure S2-a(3-1,3-2)) and 850 °C (Figure S2-a(4-1,4-2)), respectively. There were spheres (0.7–0.8 μm) and filaments (60–130 nm) of carbon material formation at 650–850 °C (Figure S2-b).

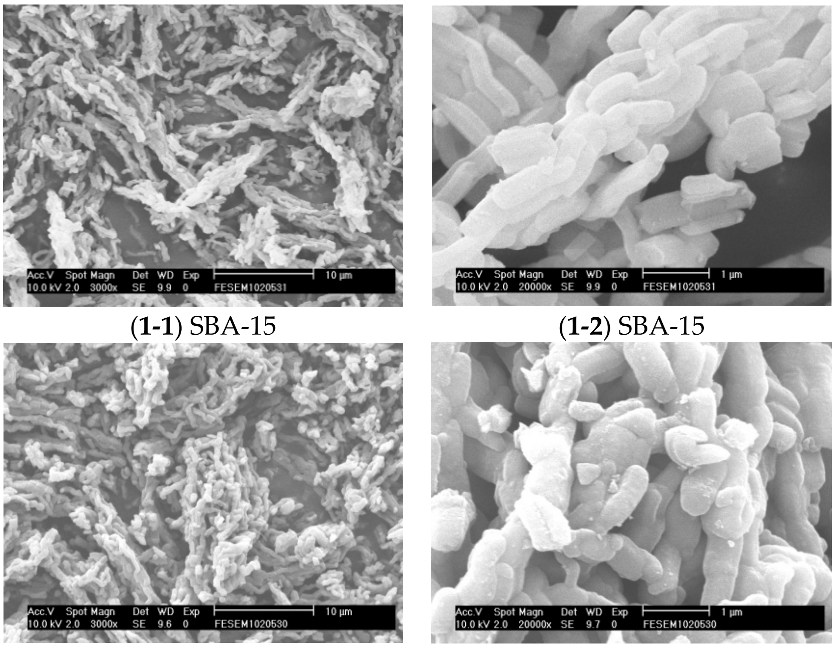

(**1-1**) SBA-15 (**1-2**) SBA-15

(**2-1**) SBA-650 (**2-2**) SBA-650

Figure S1-a. *Cont.*

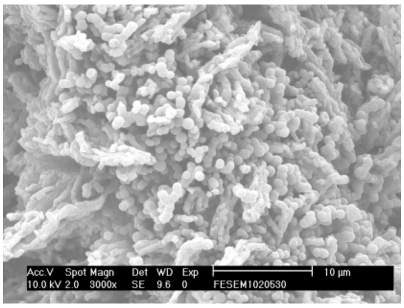

(**3-1**) SBA-750

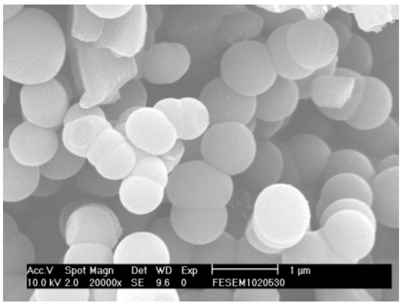

(**3-2**) SBA-750

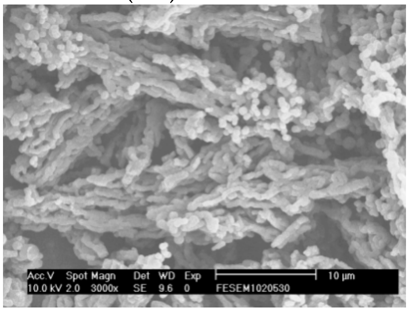

(**4-1**) SBA-850

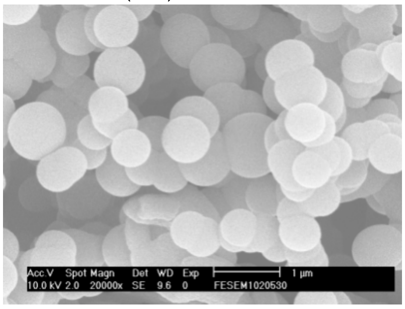

(**4-2**) SBA-850

Figure S1-a. SEM photos of SBA-15, and carbon material formation on SBA-15.

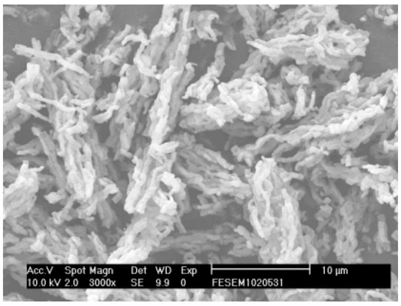

(**1-1**) Ni-SBA-15

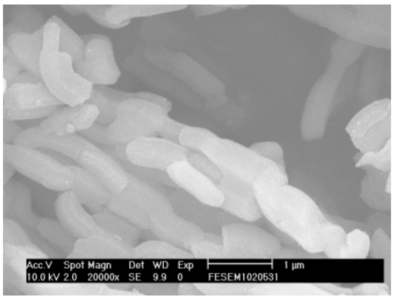

(**1-2**) Ni-SBA-15

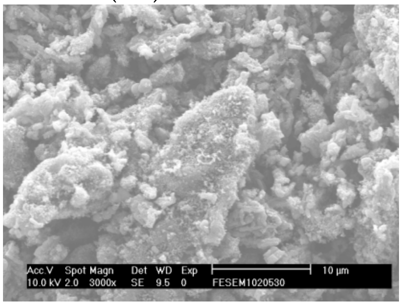

(**2-1**) Ni-SBA-650

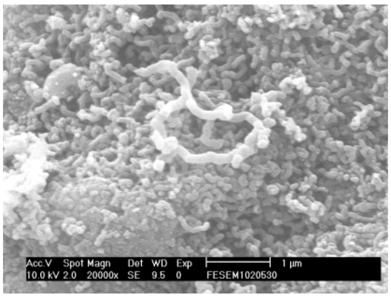

(**2-2**) Ni-SBA-650

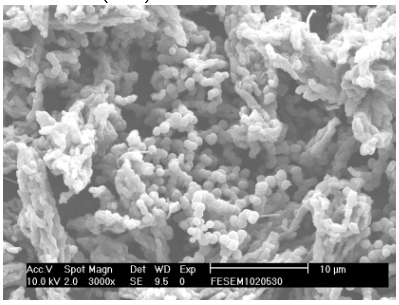

(**3-1**) Ni-SBA-750

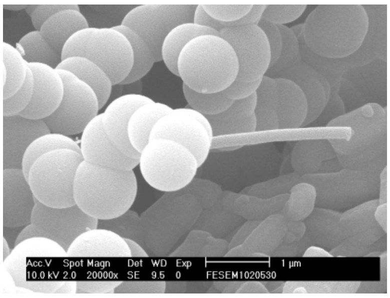

(**3-2**) Ni-SBA-750

Figure S1-b. *Cont.*

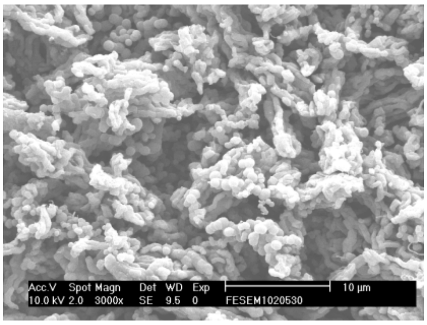

(**4-1**) Ni-SBA-850

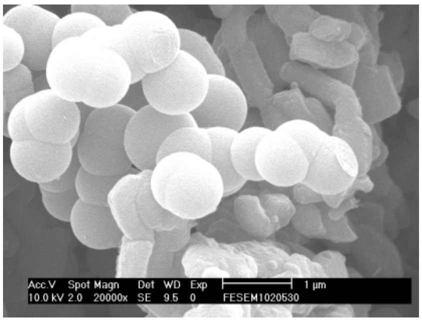

(**4-2**) Ni-SBA-850

Figure S1-b. SEM photos of Ni-SBA-15 and carbon material formation on Ni-SBA-15.

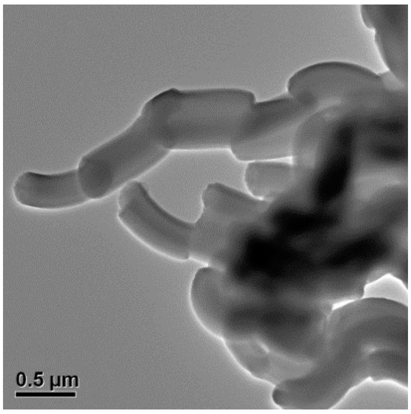

(**1-1**) SBA-15

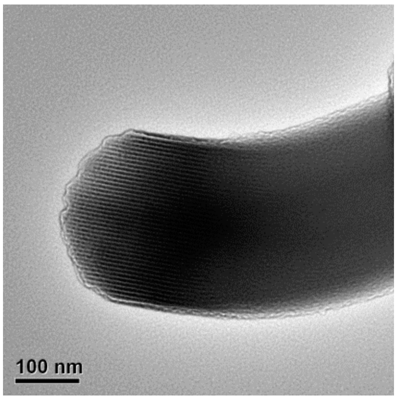

(**1-2**) SBA-15

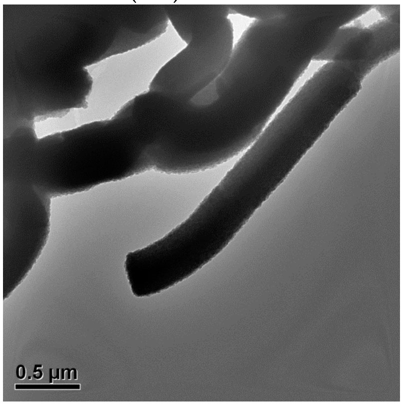

(**2-1**) SBA-650

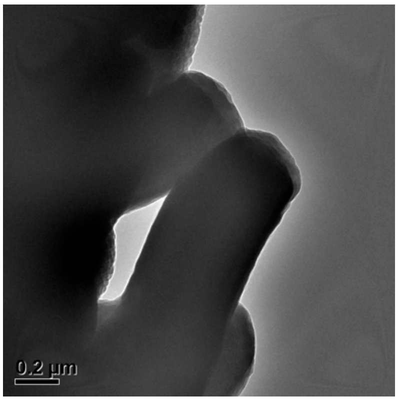

(**2-2**) SBA-650

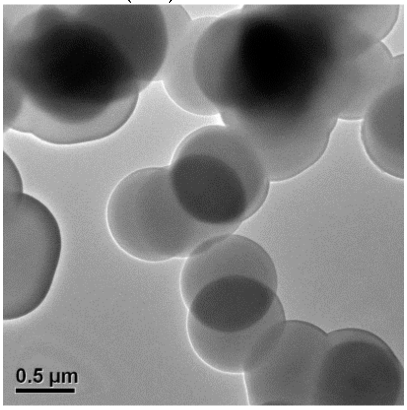

(**3-1**) SBA-750

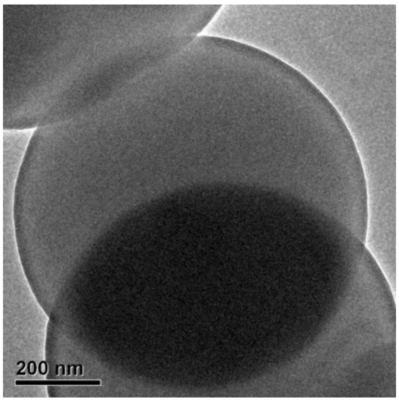

(**3-2**) SBA-750

Figure S2-a. *Cont.*

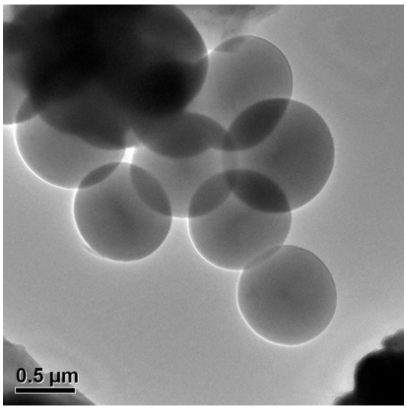

(**4-1**) SBA-850

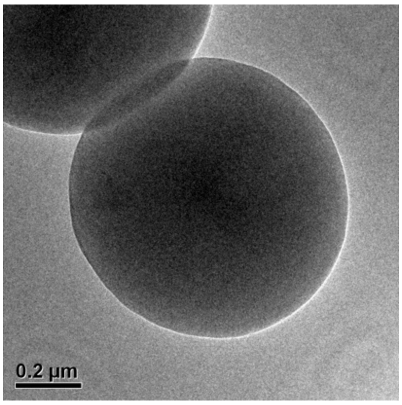

(**4-2**) SBA-850

Figure S2-a. TEM photos of SBA-15, and carbon material formation on SBA-15.

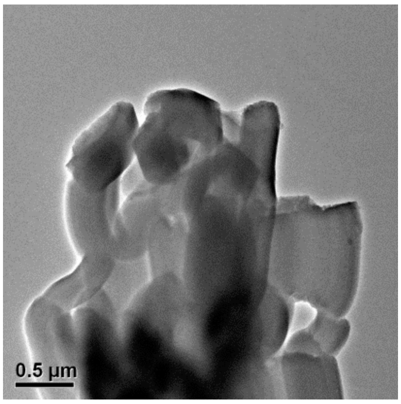

(**1-1**) Ni-SBA-15

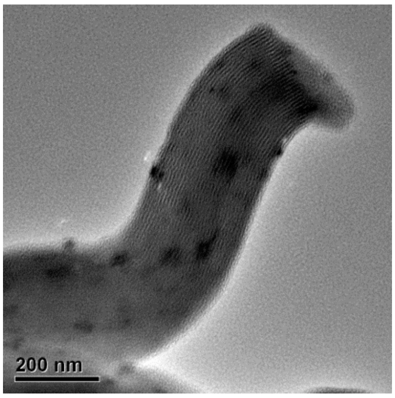

(**1-2**) Ni-SBA-15

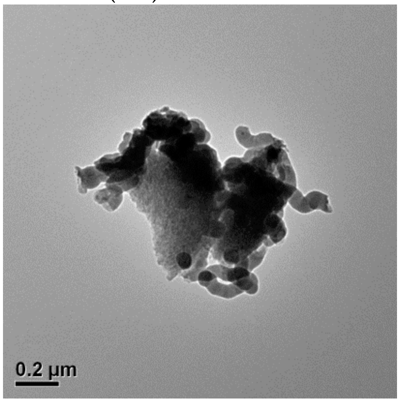

(**2-1**) Ni-SBA-650

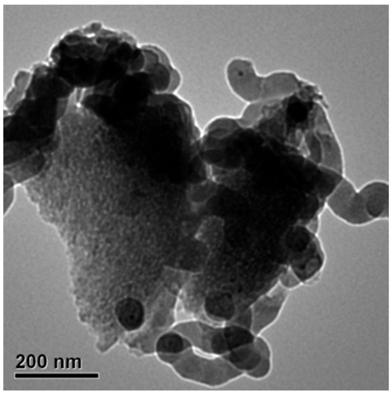

(**2-2**) Ni-SBA-650

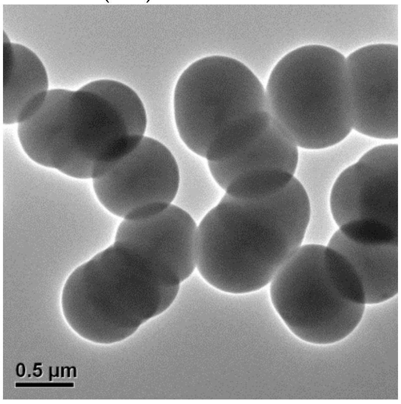

(**3-1**) Ni-SBA-750

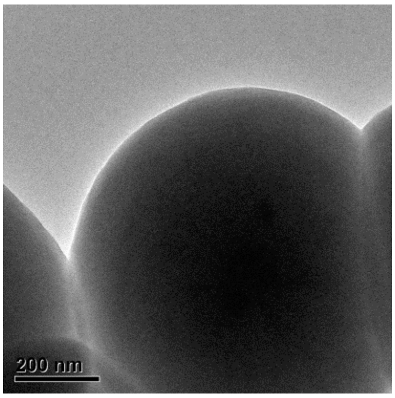

(**3-2**) Ni-SBA-750

Figure S2-b. *Cont.*

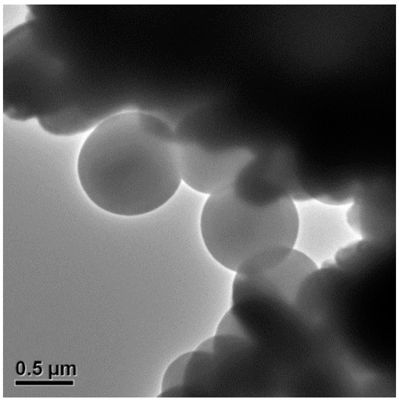

(**4-1**) Ni-SBA-850

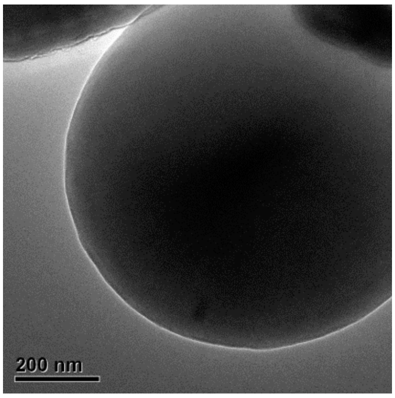

(**4-2**) Ni-SBA-850

Figure S2-b. TEM photos of Ni-SBA-15 and carbon material formation on Ni-SBA-15.
